# Flare phenomenon visualized by ^99m^Tc-bone scintigraphy has prognostic value for patients with metastatic castration-resistant prostate cancer

**DOI:** 10.1007/s12149-024-01914-8

**Published:** 2024-03-13

**Authors:** Xue Zhang, Kenichi Nakajima, Atsushi Mizokami, Hiroyuki Horikoshi, Koshiro Nishimoto, Katsuyoshi Hashine, Hideyasu Matsuyama, Satoru Takahashi, Hiroshi Wakabayashi, Seigo Kinuya

**Affiliations:** 1 Department of Nuclear Medicine, Kanazawa University, Kanazawa, Japan; 2 Department of Functional Imaging and Artificial Intelligence, Kanazawa University, 13-1 Takara-machi, Kanazawa, 920-8640 Japan; 3 Department of Urology, Kanazawa University, Kanazawa, Japan; 4 Department of Diagnostic Radiology, Gunma Prefectural Cancer Center, Ota, Japan; 5 Department of Uro-Oncology, Saitama Medical University International Medical Center, Saitama, Japan; 6 Department of Urology, Faculty of Medicine, University of Miyazaki, Miyazaki, Japan; 7 Department of Urology, NHO Shikoku Cancer Center, Matsuyama, Japan; 8 Department of Urology, JA Yamaguchi Kouseiren Nagato General Hospital, Nagato, Japan; 9 Department of Urology, Nihon University School of Medicine, Tokyo, Japan

**Keywords:** Bone scan index, Chemotherapy, Multicenter study, Prognosis, Biomarker

## Abstract

**Objective:**

This study aimed to determine the prognostic value of the flare phenomenon in patients with metastatic castration-resistant prostate cancer (mCRPC) using the bone scan index (BSI) derived from ^99m^Tc-methylenediphosphonate (MDP) bone scintigraphy images.

**Methods:**

We categorized 72 patients from the PROSTAT-BSI registry with mCRPC who were followed-up for 2 years after starting docetaxel chemotherapy to groups based on pre-chemotherapy BSI values of < 1, 1–4, and > 4. We assessed the effects of the flare phenomenon (defined as a > 10% increase in the BSI within 3 months of starting chemotherapy, followed by > 10% improvement within the next 3 months) on survival using Kaplan–Meier curves and Cox proportional hazard analyses.

**Results:**

The flare phenomenon was found in 26 (36%) of the 72 patients. Prostate-specific antigen (PSA), alkaline phosphatase (ALP), and hemoglobin (Hb) levels steadily increased, then deteriorated in patients with and without flare, respectively. Elevated BSI and PSA values at 3 months after starting therapy and the absence of abiraterone or/and enzalutamide therapy led to poor 2-year overall survival (OS) in the group without flare. In contrast, no influence was noticeable in the group with flare. The results of multivariable analyses that included only factors associated with PSA and BSI showed that increased baseline BSI (hazard ratio [HR], 1.39; 95% confidence interval [CI], 1.04–1.86; *P* = 0.023) and PSA (HR, 7.15; 95% CI 2.13–24.04; *P* = 0.0015) values could be independent risk factors for patients with mCRPC without flare. However, these factors lost significance during flare. The risk for all-cause death was significantly higher among patients with BSI > 4 without, than with flare. The results of univariable analyses indicated that flare positively impacted survival (HR, 0.24; 95% CI 0.06‒0.91; *P* = 0.035). Multivariable analysis did not identify any factors that could predict outcomes.

**Conclusion:**

Favorable prognosis, with fewer disturbances from other factors such as the use of abiraterone or/and enzalutamide, PSA changes, and BSI, was attainable in cases when the mCRPC patient demonstrated flare phenomenon. Follow-up bone scintigraphy at least every 3 months could help to determine the prognosis of patients with bone metastasis of mCRPC.

## Introduction

Prostate cancer is the second most prevalent type of malignancy among men, with a mortality rate of 6.8% and a particularly high prevalence in Americas, Oceania, most parts in Africa, western Europe, and Japan [1]. About 10‒15 months after standard androgen deprivation therapy (ADT), most prostate tumors become castration resistant and lead to increased risk of death and metastasis [2]. Bone metastases develop in ~ 90% of patients with castration-resistant prostate cancer (CRPC) during therapy, and 33% develop within 2 years [3]. Therefore, prompt detection, severity assessment, and therapy response monitoring of bone lesions are critically important.

The bone scan index (BSI) is an established quantitative tool for assessing metastatic bone lesions in mCRPC and hormone-sensitive prostate cancer. It is regarded as an imaging biomarker due to its significant contribution to monitoring bone lesions and prognostic evaluations [4–7]. EXINI Bone^®^ software (EXINI Diagnostics, Lund, Sweden) is the first artificial neural network to calculate BSI and it has been widely implemented in America and Europe. This software was refined as BONENAVI^®^ (FUJIFILM RI Pharma, Co. Ltd., Tokyo, Japan) to fit the Japanese demographic, and it is now generally applied in Japan [8, 9].

The bone flare phenomenon (flare) is defined as an initial improvement after therapy followed by apparent progression identified by bone scintigraphy [10]. Although generally accepted as a healing or reactive process, the underlying mechanisms of flare remain unclear. The incidence of this phenomenon is relatively low, but is nevertheless controversial [11].

Our multicenter PROSTAT-BSI study previously investigated the prognostic value of BSI in patients with prostate cancer treated by chemotherapy and standard hormonal therapy [12]. The BSI was recognized as a potential predictor of a poor prognosis of both mCRPC and metastatic hormone-sensitive prostate cancer (mHSPC). Flare was identified in some patients, but we did not elucidate its role and prognostic significance [12].

Thus, we speculated that flare is a bone metabolic response to chemotherapy that could serve as an indicator of a more favorable prognosis in patients with mCRPC. Therefore, the present study aimed to determine the prognostic value of the flare phenomenon determined by bone scintigraphy based on the BSI in patients with mCRPC.

## Materials and methods

### Patients

A cohort of patients with confirmed bone mCRPC (*n* = 72) was sourced from several institutions included in the PROSTAT-BSI registry. The patients were monitored for 2 years from the start of docetaxel chemotherapy based on the mCRPC mortality rate from our previous findings [12] regardless of endpoint (all-cause death) evaluation. Biopsy samples were assessed at the time of prostate cancer diagnosis using Gleason scores (GS). Baseline values for serological biomarkers prostate‐specific antigen (PSA), alkaline phosphatase (ALP), bone alkaline phosphatase (BAP), cross‐linked telopeptide parts of type I collagen (1-CTP), and hemoglobin (Hb) as well as the BSI were measured immediately before docetaxel chemotherapy (month 0). These values were assessed every 3 months in the first year followed by the timepoints of 1 and 2 years. Disease progression was determined based on PSA values.

### Bone scintigraphy

The status of bone metastasis was assessed by ^99m^Tc-methylene diphosphonate (MDP) whole-body bone scintigraphy on the 3rd, 6th, 9th, 12th, and 24th month of starting docetaxel chemotherapy. Whole lesion BSI was calculated using BONENAVI^®^ software (FUJIFILM Toyama Chemical/PDRadiophama, Inc., Tokyo, Japan), and the artificial neural network-based algorithm as described [9, 13]. Based on the BSI before starting chemotherapy, patients were assigned to 3 groups; BSI < 1, 1 to < 4, and BSI > 4 as described in PROSTAT-BSI [12]. Flare was defined as an increase in the BSI of > 10% at 3 months after starting docetaxel, followed by a further > 10% improvement over the next 3 months.

### Statistical analysis

Continuous variables are shown as means ± standard deviation. Categorical variables are shown as proportions (%). Changes in BSI, PSA, ALP, BAP, 1-CTP, and Hb were normalized by comparison with baseline values at month 0. Differences among groups were analyzed using *t* tests, Kruskal–Wallis tests, and Pearson correlation coefficients. Changes in PSA and Hb values between before and after therapy were assessed using paired *t* tests. Survival functions were estimated using Kaplan–Meier curves with log-rank and Wilcoxon tests. Potential risk factors associated with mortality were identified using Cox proportional hazards models. All data were statistically analyzed using JMP 17 (SAS Institute, Cary, NC, USA). Values with *P* < 0.05 were considered significant.

## Results

### Patients’ characteristics

Table 1 shows the characteristics of the patients. By the end of the 2-year observation period, 27 (38%) of the 72 patients had died. Among them, 20 (74%) were due to prostate cancer. The mean overall (OS) and progression-free survival (PFS) were 19.8 and 10.3 months, respectively, and 39 (54%) of the patients were treated with abiraterone or/and enzalutamide. Overall, 26 (36%) of 72 patients had flare based on changes in the BSI.

**Table 1:** Characteristics of patients

Variable	Total	With flare	Without flare	*P*
Patients (*n*)	72	26 (36%)	46 (64%)	
Age (years)	70.8 ± 7.4	67.0 ± 6.6	71.8 ± 7.6	0.12
Overall survival (m)	19.8 ± 0.7	21.0 ± 1.0	19.0 ± 1.0	0.11
Progression-free survival (m)	10.3 ± 0.9	10.4 ± 1.5	10.2 ± 1.1	0.79
**Events**				
All-cause death	27 (38%)	7 (27%)	20 (44%)	0.16
Prostate cancer death	20 (28%)	7 (27%)	13 (28%)	0.90
Non-regional LN metastases	21 (30%)	8 (31%)	13 (30%)	0.96
Lung/liver metastasis	10 (14%)	4 (15%)	6 (13%)	0.78
Gleason score ≥ 9	42 (63%)	17 (74%)	25 (57%)	0.17
**Baseline biomarkers**				
PSA (ng/mL)	114.0 ± 228.6	178.6 ± 321.8	77.5 ± 145.3	0.32
ALP (IU/mL)	564.0 ± 706.8	505.5 ± 492.6	597.1 ± 806.4	0.82
BAP (μg/L)	51.4 ± 78.5	48.6 ± 57.4	53.0 ± 88.5	0.51
1-CTP (ng/mL)	7.9 ± 8.8	6.9 ± 3.5	8.4 ± 10.5	0.71
Hb (g/dL)	11.8 ± 1.5	11.9 ± 1.2	11.8 ± 1.7	0.98
CRP (mg/dL)	1.3 ± 2.8	1.5 ± 2.7	1.2 ± 2.8	0.33
BSI (%)	3.2 ± 3.0	3.4 ± 3.1	3.1 ± 2.9	0.60
**Number of patients with increased biomarker after 3 months of therapy**				
PSA	25 (35%)	6 (24%)	19 (41%)	0.14
ALP	13 (18%)	6 (24%)	7 (15%)	0.36
BAP	10 (17%)	4 (19%)	6 (15%)	0.72
1-CTP	11 (19%)	5 (28%)	6 (15%)	0.27
Hb	23 (32%)	13 (52%)	10 (22%)	0.0093
**Number of patients with increased biomarker after 2 years of therapy**				
PSA	46 (64%)	13 (50%)	33 (72%)	0.065
ALP	38 (53%)	13 (50%)	25 (54%)	0.72
BAP	23 (37%)	10 (45%)	13 (32%)	0.28
1-CTP	40 (63%)	15 (71%)	25 (60%)	0.35
Hb	15 (20.8%)	11 (42%)	4 (9%)	0.0007
**Therapy**				
Abiraterone/enzalutamide	39 (54%)	17 (65%)	22 (48%)	0.15
Radiation therapy	13 (18%)	4 (15%)	9 (20%)	0.66

*1-CTP* cross‐linked telopeptide parts of type I collagen, *ALP* alkaline phosphatase, *BAP* bone alkaline phosphatase, *CRP* C‐reactive protein, *Hb* haemoglobin, *LN* lymph node, *m* months, *PSA* prostate‐specific antigen, *y* years

### Relationships between flare phenomenon and other factors

**Fig. 1:**
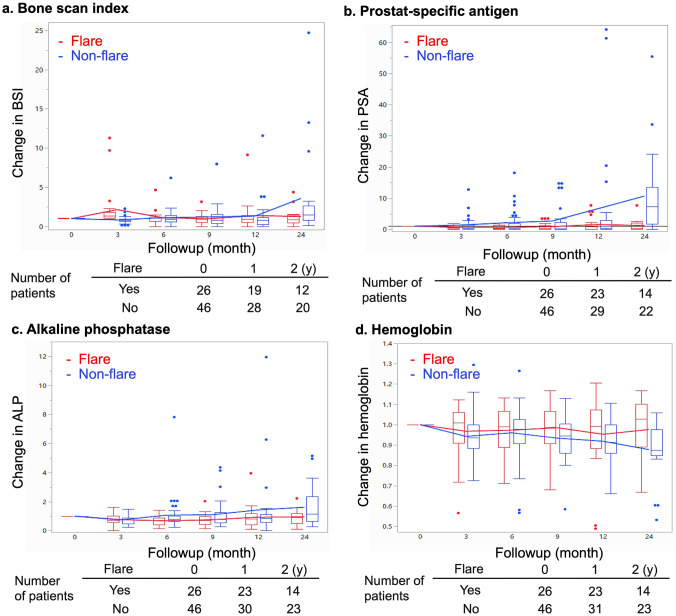
Serial changes in  BSI (**a**), PSA (**b**), ALP (**c**), and hemoglobin (**d**) during follow-up. Ratios compared with the baseline condition are shown, *ALP* alkaline phosphatase, *BSI* bone scan index, *PSA* prostate-specific antigen; red and blue fonts, with and without flare, respectively

**Fig. 2:**
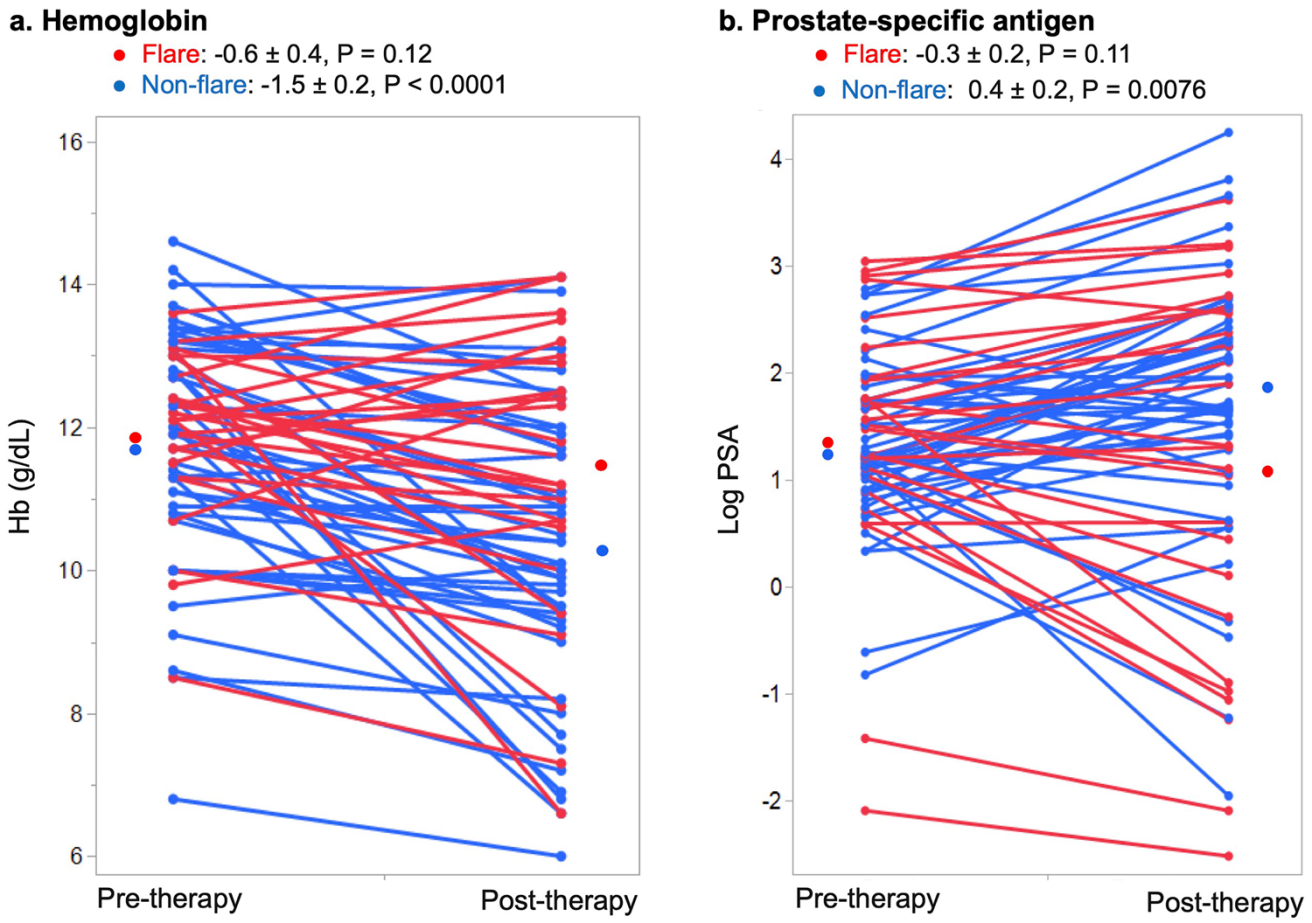
Changes in Hb (**a**) and PSA (**b**) between before and after therapy. Each line in the parallel coordinate plot shows data derived from a single patient before and after therapy. With (red) and without (blue) flare: Hb, − 0.6 ± 0.4, *P* = 0.12 and − 1.5 ± 0.2, *P* < 0.0001, respectively; PSA, − 0.3 ± 0.2, *P* = 0.11 and 0.4 ± 0.2, *P* = 0.0076, respectively. Red and blue dots in left and right panels indicate mean Hb and PSA values, respectively. *Hb* hemoglobin, *PSA* prostate-specific antigen

Figure 1 shows changes in the rates of BSI, PSA, ALP, and Hb over time in patients with and without flare, respectively. According to the flare concept, an obvious upward and downward tendency during the first 6 months was observed in the flare group and become stabilized without significant elevation thereafter. In contrast, the BSI in the group without flare briefly decreased at the end of month 3, then gradually increased and surpassed that of the flare group after 6 months of treatment (Fig. 1a). Overall, levels of PSA, ALP, and Hb did not notably change in the group with flare during the 2-year observation period, whereas PSA and ALP tended to increase and Hb tended to decrease in the group without flare (Fig. 1b–d). Figure 2 shows changes between pre- and post-therapy in PSA and Hb. Neither PSA nor Hb obviously differed between the pre- and post-therapy periods for patients with flare, but both were significantly exacerbated in patients without flare. Hemoglobin levels improved in 4 (9%) of 46 vs. 11 (42%) of 26 patients (*P* = 0.0007). without and with flare, respectively However, even though PSA and ALP similarly changed, the difference did not reach statistical significance (Table 1).

Table 2 shows the characteristics of the subgroups based on BSI. Gleason scores ≥ 9 and BSI positively correlated with borderline significance (*P* = 0.053) without flare, compared with an apparently opposite trend with flare, although the difference did not reach significance. All patients with a higher BSI tended to have worse baseline blood test results, regardless of the flare phenomenon. However, levels of ALP (*P* = 0.004) and BAP (*P* = 0.0021) in the group with flare, and of ALP (*P* = 0.0007), BAP (*P* = 0.0019), 1-CTP (*P* = 0.0315), and Hb (*P* = 0.0184) in the group without flare significantly differed. The incidence of radiation therapy, including external palliative radiation and internal radiation with strontium-89 and radium-223, did not show significant differences between the flare and non-flare groups (15 vs. 20%, *P* = 0.66). The rate of abiraterone or/and enzalutamide administration was not affected by flare or the BSI.

**Table 2:** Characteristics of patients sub-grouped according to BSI

Variable	With flare			*P*	Without flare			*P*
BSI	< 1	1‒4	> 4		< 1	1‒4	> 4	
Patients (*n*)	7	7	12		15	20	11	
Age (years)	68.9 ± 3.7	69.9 ± 11.0	68.5 ± 5.1	0.92	69.4 ± 6.1	74.1 ± 8.5	71.0 ± 7.1	0.19
**Events**								
All-cause death	2 (29%)	2 (29%)	3 (25%)	0.98	3 (20%)	9 (45%)	8 (73%)	0.03
Prostate cancer death	2 (29%)	2 (29%)	3 (25%)	0.98	2 (13%)	4 (20%)	7 (64%)	0.01
Non-regional LN metastases	3 (43%)	2 (29%)	3 (25%)	0.71	4 (27%)	3 (18%)	6 (55%)	0.11
Lung/liver metastasis	7 (100%)	7 (100%)	8 (67%)	0.06	15 (100%)	17 (85%)	8 (73%)	0.12
Gleason score ≥ 9	6 (86%)	5 (83%)	6 (60%)	0.41	5 (33%)	12 (63%)	8 (80%)	0.053
**Baseline biomarkers**								
PSA (ng/mL)	18.6 ± 14.5	184.7 ± 409.2	268.4 ± 344.7	0.09	51.1 ± 135.1	81.7 ± 135.8	105.9 ± 180.0	0.11
ALP (IU/mL)	206.9 ± 39.5	317.9 ± 171.3	789.1 ± 606.4	0.004	251.2 ± 107.1	344.4 ± 177.5	1528.2 ± 1260.9	0.0007
BAP (μg/L)	11.2 ± 4.2	19.8 ± 10.7	79.2 ± 66.1	0.0021	16.9 ± 13.2	27.1 ± 23.2	149.1 ± 144.4	0.0019
1-CTP (ng/mL)	4.9 ± 4.1	6.9 ± 2.8	7.7 ± 3.4	0.18	5.1 ± 2.5	6.1 ± 2.5	17.3 ± 19.4	0.0315
Hb (g/dL)	11.8 ± 1.1	11.9 ± 1.8	12.0 ± 0.8	0.65	12.6 ± 1.2	11.8 ± 1.3	10.6 ± 2.3	0.0184
CRP (mg/dL)	1.7 ± 3.2	2.1 ± 3.8	0.9 ± 1.2	0.76	0.3 ± 0.3	0.9 ± 2.3	3.3 ± 4.8	0.37
**Number of patients with increased biomarker after 3 months of therapy**								
PSA	0 (0%)	2 (29%)	4 (33%)	0.28	9 (60%)	6 (30%)	4 (36%)	0.19
ALP	3 (50%)	1 (14%)	2 (17%)	0.23	3 (20%)	4 (20%)	0 (0%)	0.27
BAP	2 (33%)	0 (0%)	2 (18%)	0.42	4 (31%)	2 (12%)	0 (0%)	0.12
1-CTP	2 (50%)	0 (0%)	3 (30%)	0.28	4 (31%)	2 (12%)	0 (0%)	0.12
Hb	3 (50%)	4 (57%)	6 (50%)	0.95	3 (20%)	4 (20%)	3 (27%)	0.88
**Therapy**								
Abiraterone/enzalutamide	5 (71%)	4 (57%)	8 (67%)	0.85	9 (60%)	8 (40%)	5 (45%)	0.49

*1-CTP* cross‐linked telopeptide parts of type I collagen, *ALP* alkaline phosphatase, *BAP* bone alkaline phosphatase, *CRP* C‐reactive protein, *Hb* haemoglobin, *LN* lymph node, *PSA* prostate‐specific antigen

### Survival analysis

**Fig. 3:**
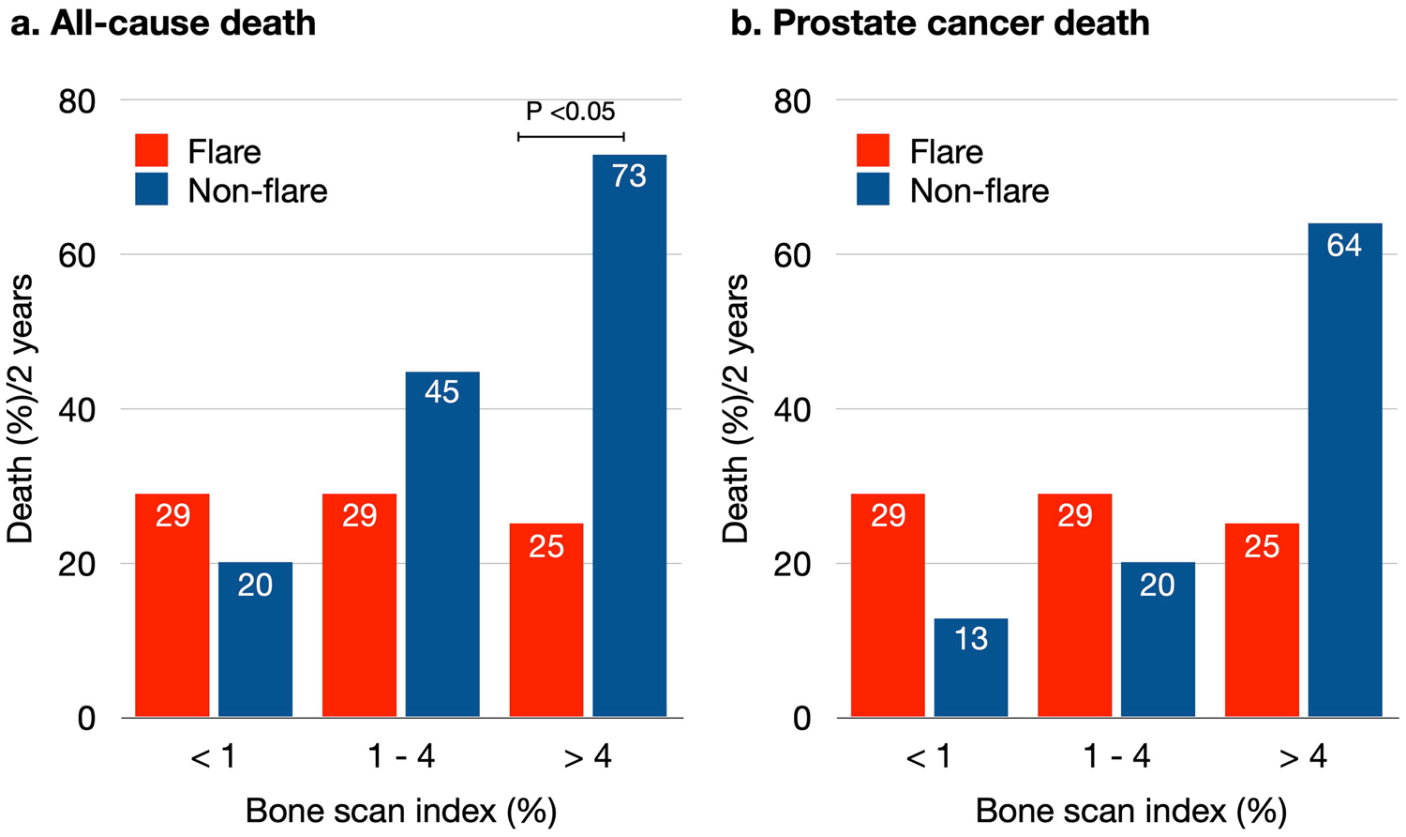
Contingency comparison of death due to all-causes (left) and prostate cancer (right) for 2 years in patients with (red) and without (blue) flare

We compared the survival rates of patients who died of prostate cancer and of all causes. Prostate cancer was the sole cause of death among 7 (27%) of 26 patients with flare during 2 years of monitoring. Seven (35%) of twenty patients in the group without flare died of causes unrelated to prostate cancer. Although overall mortality did not significantly differ between these groups, the outcomes within the BSI subgroups indicated that the mortality rate remained consistent among patients with flare. A higher BSI was associated with increased all-cause (*P* = 0.03) and prostate cancer-associated (*P* = 0.01) death rates in the group without flare. The incidence of death was higher in patients without flare and with BSI > 4 (73 and 64% for all-cause and for prostate cancer-related causes). In contrast, all-cause and prostate cancer-related mortality was significantly lower among patients with flare within the same BSI subgroup (25%; *P* = 0.0221; Fig. 3).

**Fig. 4:**
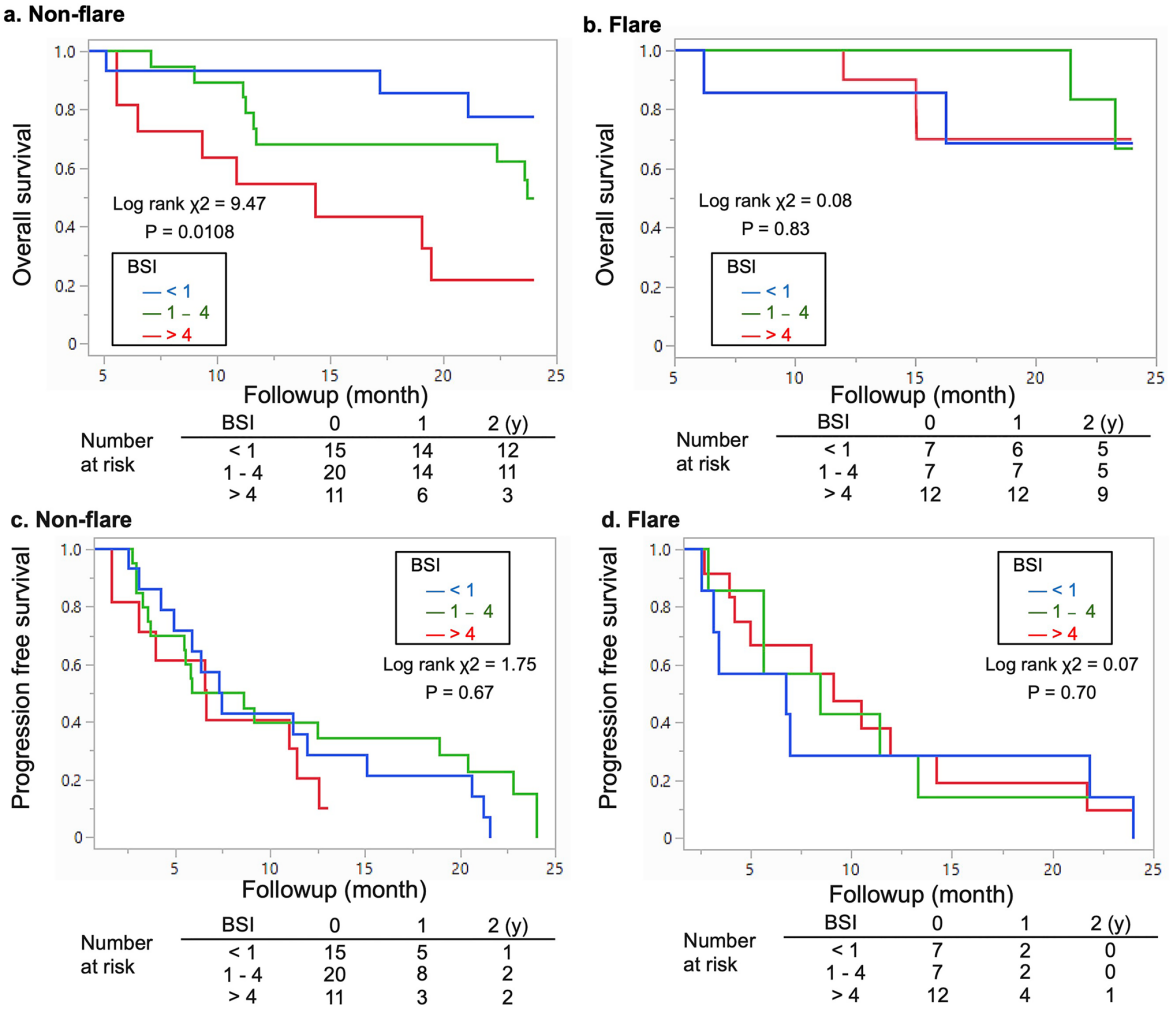
Overall and progression-free survival of patients. Overall survival without (**a**) and with (**b**) flare. Progression-free survival without (**c**) and with (**d**) flare according to BSI < 1 (blue), 1‒4 (green), and > 4 (red). *BSI* bone scan index, *m* months

The trend in OS was similar. The OS did not significantly differ among BSI subgroups with flare, and none of the patients reached the median OS within 2 years. An elevated BSI was linked to shorter OS in patients without flare. The median OS was reached in 23.7 and 14.3 months by BSI 1‒4 and > 4 subgroups, respectively, but was never achieved by the subgroup with BSI < 1 (Fig. 4a, b). The PFS judged by PSA did not change regardless of flare or the BSI (Fig. 4c, d).

**Fig. 5:**
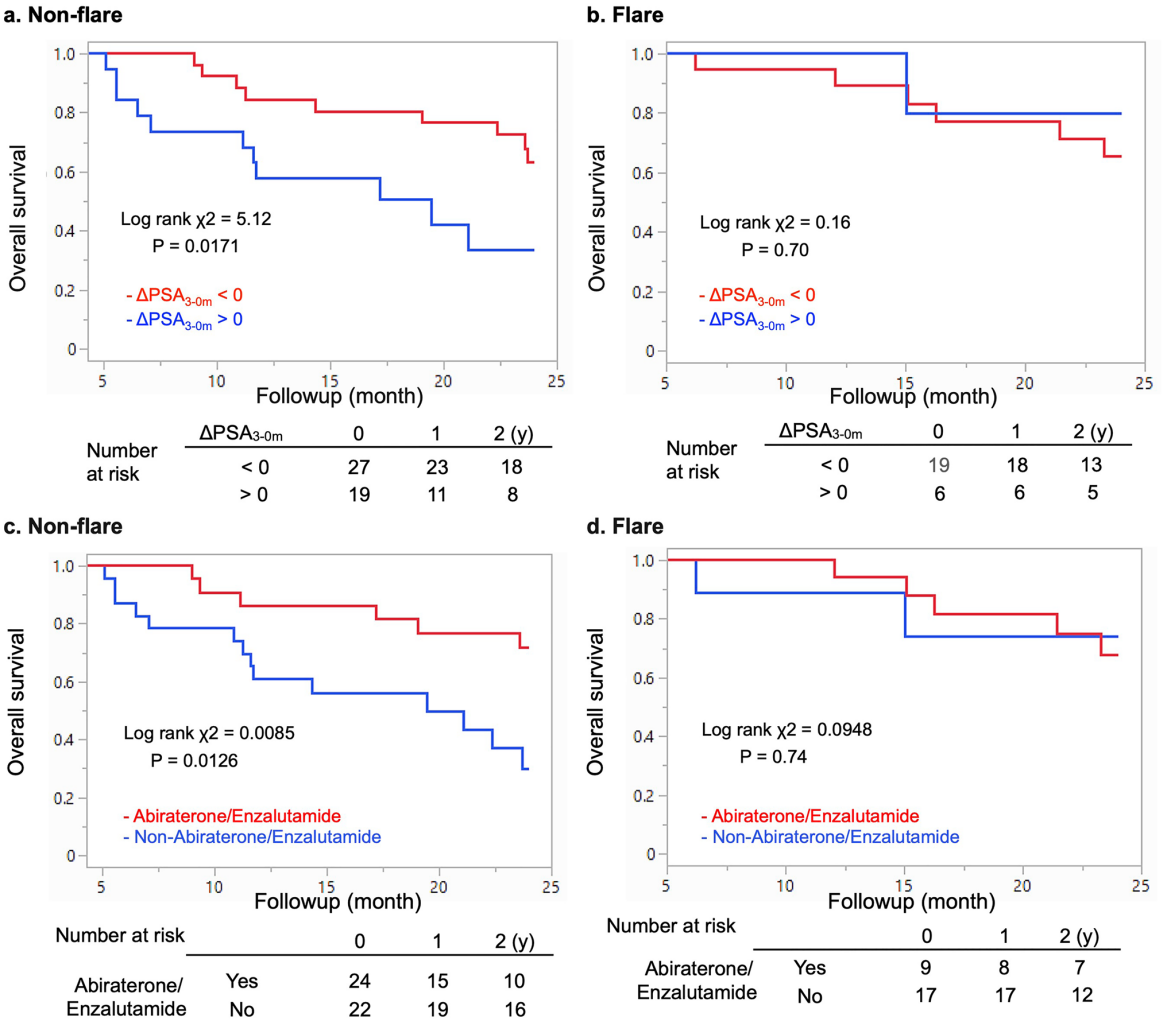
Overall survival of patients without (**a**) and with (**b**) flare. Overall survival without (**a**, **c**) and with (**b**, **d**) flare. Binary based on changes in PSA (**a**, **b**) and abiraterone/enzalutamide (**c**, **d**)

We compared the impact of blood biomarkers on the survival of patients with and without flare. We found that elevated PSA at month 3 did not affect the survival of patients with flare, but predicted a poor prognosis for those without flare (Fig. 5a, b). The outcomes of abiraterone and/or enzalutamide therapy were similar in the group with flare, whereas the OS might be significantly longer among patients without flare (Fig. 5c, d).

### Prediction of overall survival

Table 3 shows the results of Cox proportional hazards models. The univariable results showed that baseline ALP (*P* < 0.0001), BAP (*P* = 0.0001), 1-CTP (*P* = 0.0023), Hb (*P* = 0.0002), CRP (*P* = 0.0116), and BSI (*P* = 0.0082) were significantly associated with OS in patients without flare. Elevated PSA after 3 months of therapy (*P* = 0.0294) and BSI > 4 (*P* = 0.0082) were also significantly associated with 2-year OS in this group. Liver or lung metastasis (*P* = 0.0154) was the sole significant factor in the group with flare. The multivariable analysis of PSA- and BSI-related factors indicated that baseline BSI (*P* = 0.0233) and elevated PSA (*P* = 0.0015) could be considered as independent risk factors for patients with mCRPC without flare. However, the significance of all factors was lost during flare.

**Table 3:** Findings of univariable and multivariable proportional hazards analysis

Variable	With flare		Without flare	
	HR (95% CI)	*P*	HR (95% CI)	*P*
**Univariable analysis**				
Age (years)	0.972 (0.878‒1.086)	0.60	1.045 (0.986‒ 1.108)	0.14
**Events**	0.60 (0.986‒1.045)			
Non-regional LN metastases	0.727 (0.141‒3.758)	0.70	1.306 (0.489‒3.488)	0.59
Lung/liver metastasis	6.732 (1.440‒31.470)	0.0154	1.145 (0.335‒3.910)	0.83
Gleason score ≥ 9	1.835 (0.214‒15.740)	0.58	1.002 (0.394‒2.545)	0.99
Baseline BSI (%)	0.904 (0.633‒1.163)	0.50	1.188 (1.038‒1.345)	0.0082
BSI > 4 (%)	1.081 (0.241‒4.843)	0.92	3.406 (1.373‒8.453)	0.0082
**Baseline biomarkers**				
PSA (ng/mL)	0.996 (0.981‒1.001)	0.40	1.001 (0.997‒1.003)	0.60
ALP (IU/mL)	1.000 (0.999‒1.001)	0.45	1.001 (1.000‒1.001)	< 0.0001
BAP (μg/L)	1.002 (0.986‒1.012)	0.77	1.009 (1.004‒1.014)	0.0001
1-CTP (ng/mL)	1.135 (0.851‒1.490)	0.35	1.045 (1.012‒1.073)	0.0023
Hemoglobin (Hb) (g/dL)	0.879 (0.497‒1.823)	0.69	0.613 (0.475‒0.792)	0.0002
CRP (mg/dL)	1.081 (0.717‒1.391)	0.61	1.191 (1.021‒1.354)	0.0116
**Increased biomarker after 3 months**				
PSA	0.649 (0.077‒5.436)	0.69	2.709 (1.105‒6.642)	0.0294
ALP	1.914 (0.367‒9.988)	0.44	0.712 (0.162‒3.085)	0.65
BAP	2.708 (0.493‒14.879)	0.25	0.297 (0.039‒2.240)	0.24
1-CTP	1.460 (0.149‒14.265)	0.75	1.281 (0.365‒4.491)	0.70
Hb	1.417 (0.316‒6.353)	0.65	0.532 (0.156‒1.817	0.31
**Therapy**				
Abiraterone/enzalutamide	0.946 (0.183‒4.898)	0.95	0.293 (0.111‒0.771)	0.0130
**Multivariable analysis of BSI and PSA**				
PSA (ng/mL)	0.995 (0.973‒1.001)	0.35	1.000 (0.997‒1.003)	0.95
Increased PSA	0.479 (0.143‒1.086)	0.46	7.150 (2.127‒24.039)	0.0015
BSI (%)	0.798 (0.471‒1.211)	0.13	1.392 (1.038‒1.855)	0.0233
BSI > 4 (%)	29.813 (0.517‒1720.041)	0.10	0.861 (0.145‒5.105)	0.87

*1-CTP* cross‐linked telopeptide parts of type I collagen, *ALP* alkaline phosphatase, *BAP* bone alkaline phosphatase, *BSI* bone scan index, *CI* confidence internal (lower to upper 95%), *CRP* C‐reactive protein, *Hb* haemoglobin, *HR* hazard ratio (range), *LN* lymph node, *PSA* prostate‐specific antigen

The prognosis was poor for patients with BSI > 4. Therefore, we investigated predictors of death using Cox proportional hazards models. The univariate analysis selected flare (*P* = 0.0353), high baseline Hb (*P* = 0.0190) and therapy with abiraterone or/and enzalutamide (*P* = 0.0165) as significant predictors of a longer OS. Conversely, higher ALP (*P* = 0.0033), BAP (*P* = 0.0051), and 1-CTP (*P* = 0.0114) negatively impacted OS. However, the multivariate analysis did not select any factors that might predict outcomes (Table 4).

**Table 4:** Univariable and multivariable proportional hazards analysis of subgroup with BSI > 4

Variable	Univariable		Multivariable	
	HR (95% CI)	*P*	HR (95% CI)	*P*
Age (years)	1.074 (0.959‒1.209)	0.22		
**Event**				
Non-regional LN metastases	1.108 (0.336‒3.647)	0.87		
Lung/liver metastasis	0.912 (0.265‒3.137	0.88		
Gleason score ≥ 9	2.524 (0.531‒12.007)	0.24		
BSI (%)	1.020 (0.777‒1.302)	0.88		
Flare phenomenon	0.239 (0.063‒0.906)	0.0353	0.265 (0.052‒1.349)	0.11
**Baseline biomarkers**				
PSA (ng/mL)	0.998 (0.994‒1.001)	0.35		
ALP (IU/mL)	1.001 (1.000‒1.001)	0.0033	1.000 (0.995‒1.005	0.99
BAP (μg/L)	1.008 (1.002‒1.015)	0.0051	1.007 (0.965‒1.050	0.76
1-CTP (ng/mL)	1.046 (1.007‒1.084)	0.0114	0.995 (0.921‒1.076	0.91
Hemoglobin (Hb) (g/dL)	0.652 (0.452‒0.938)	0.0190	0.935 (0.504‒1.734	0.83
CRP (mg/dL)	1.099 (0.884‒1.305)	0.31		
**Increased biomarker after 3 months of therapy**				
PSA	2.323 (0.704‒7.670)	0.17		
ALP	1.186 (0.148‒9.524)	0.87		
BAP	0.994 (0.124‒7.975)	0.99		
1-CTP	0.924 (0.113‒7.555)	0.94		
Hb	0.524 (0.138‒1.981)	0.34		
**Therapy**				
Abiraterone/enzalutamide	0.217 (0.062‒0.757)	0.0165	0.608 (0.125‒2.957)	0.54

*1-CTP* cross‐linked telopeptide parts of type I collagen, *ALP* alkaline phosphatase, *BAP* bone alkaline phosphatase, *CI* confidence interval (lower to upper 95%), *CRP* C‐reactive protein, *Hb* haemoglobin, *LN* lymph node, *PSA* prostate‐specific antigen

## Discussion

Flare could be identified on bone scintigraphic images soon after starting various therapies. This multicenter study of patients with prostate cancer aimed to elucidate the role of this phenomenon throughout a 2-year therapeutic course. We found that flare conferred a more favorable 2-year prognosis. This suggested that bone imaging at 3 months after starting chemotherapy would help to decide appropriate management and treatment of this disease.

### Definition of flare

We defined flare based on the BSI quantified using automatic neural network-based software. Debates have been ongoing and various opinions have been offered regarding the definition of bone flare since its discovery during the 1970s. A study of 33 patients with mCRPC, treated with abiraterone, defined flare as images of deteriorating bone status at 3 months of treatment, accompanied by a 50% reduction in PSA, followed by improved bone status after 6 months of treatment [14]. Evaluations of bone lesions using ^18^F-NaF defined flare as an increased standardized uptake value (SUV) or lesion count at 6 weeks, followed by a decline at 12 weeks of therapy [15]. Here, we defined flare as a > 10% increase in the BSI at 3 months after starting docetaxel therapy, followed by an improved BSI over the next months. Our prior experience served as the foundation for this definition [12].

A longer imaging interval (16 weeks) has been recommended to avoid a potential peak of flare [7], as it could mislead treatment strategies. However, the universal consideration is that flare is evidence of osteoblastic healing or a positive response to novel hormone therapies or systemic chemotherapy [11, 16]. Therefore, we suggest that the importance of evaluating bone status by imaging at 3 months of treatment should be emphasized. An elevated BSI should be followed-up by bone imaging after 6 months to evaluate the presence or absence of flare.

### Flare and cause of mortality

The absence of flare is associated with a higher likelihood of death due to causes other than tumors. During the course of prostate cancer, 20‒40% of patients die primarily of cardiovascular and cerebrovascular diseases, and chronic obstructive pulmonary disease (COPD) that are not associated with prostate cancer. These diseases typically manifest within 5 years of being diagnosed with prostate cancer [17, 18]. None of our patients with flare died of causes other than prostate cancer, whereas 7 (35%) of 20 without flare died of such causes. The rate of prostate cancer-caused deaths was essentially equal in both groups. Anti-cancer therapy is a main factor that increases the risk of non-prostate cancer-related deaths [19–21]. This led us to speculate whether the flare phenomenon not only indicates a better response, but also suggests better individual tolerance of toxicity caused by therapy. However, further verification is needed due to the potential randomness associated with our small cohort.

### Tendencies of changes in serum biomarkers

Serum biomarkers during therapy minimally changed in patients with flare but tended to deteriorate in those without flare. Serum biomarkers play a crucial role in the early detection of cancer and in predicting the prognosis of mCRPC. In addition to PSA, bone metabolic markers, such as ALP and BAP, might also have prognostic significance for identifying patients who might derive benefits from targeted treatment of bone lesions [22–24]. Such markers are generally associated with changes in the BSI [5]. The markers PSA, ALP, and Hb tended to remain steady, and to decline in patients with and without flare, respectively. This outcome did not deviate from the general profile of BSI adjustments. Thus, we propose that these findings indicate a potentially favorable prognosis for patients with flare that might be associated with better control of bone marrow invasion.

Anemia that typically manifests as low Hb is an indicator of tumor load and the overall physiological response of patients. Hemoglobin is acknowledged as a prognostic factor in CRPC, particularly for patients treated with docetaxel [25–27]. While changes in other biomarkers after completing the entire course of therapy did not significantly differ regardless of flare, anemia progressed in 42 of 46 patients without flare. This probability was significantly higher than that of patients with flare (*P* = 0.0007) and had already emerged at 3 months of treatment. This provides a new perspective in that a bone flare not only indicates a better bone marrow response, but also indicates a significant improvement in overall systemic status. This systemic response is even sensitive to the manifestation of bone marrow reactions and could be taken into consideration when evaluating the prognosis of mCRPC.

In addition to bone, occasional ALP and PSA flares are regarded as reactions to therapy, and they are predictive of better OS and PFS [28–32]. However, whether and how ALP and PSA flare might impact the occurrence of bone flare remained unclear. We did not consider this because ALP and PSA flares typically manifest within 1‒2 months after inducing therapy [10, 28, 32], whereas we followed-up our patients every 3 months.

### BSI and prognosis

Two-year survival was positively associated with the BSI only in the group without flare. The significance of baseline BSI to survival has been investigated using various cutoff points. The BSI has been identified as an independent prognostic biomarker for patients with mCRPC treated with docetaxel (> 1 vs. ≤ 1%; *P* = 0.037). The OS was notably shorter among patients with BSI > 1% than ≤ 1% [33]. A large phase III assessment also established a connection between a higher BSI and a worse OS [34]. A meta-analysis substantially linked a higher baseline BSI with a poorer OS (*P* = 0.007), particularly in an Asian population [6]. Nonetheless, a significant correlation has not been found between the baseline BSI and the prognosis of patients with mCRPC [12, 35]. We found quite different manifestations depending on the subgroups with flare. The 2-year OS was almost identical between patients with flare and a significantly high or low BSI, but was positively associated with the BSI in the group without flare. This might explain the diversity of outcomes associated with BSI and prognosis. Besides, our univariable analysis indicated that flare might diminish the probability of mortality in patients with BSI > 4. Despite being particularly rare, investigations into the relationship between bone flare and prognosis have resulted in contradictory conclusions. A secondary analysis of the PREVAIL and AFFIRM randomized clinical trials concluded that flare leads to a reduced OS in patients with mCRPC treated with docetaxel, even though the time to PSA progression and the secondary endpoints of PFS did not significantly differ [36]. Another study found no impact of flare on OS and PFS [11]. However, small cohorts and the specificity of administered drugs have been insufficient to generate concrete conclusions. We analyzed death from all causes and specifically from prostate cancer. Both of elevated all-cause death (*P* = 0.03) and mortality attributed to prostate cancer rates were correlated with increasing BSI (*P* = 0.01) in the absence of flare. This association was not evident in the flare group. All-cause mortality of BSI > 4 subgroup was significantly higher among patients without, than with flare (*P* = 0.0221).

### Prognostic effects of flare combined with other variables

Some factors that impact prognosis such as serum biomarkers and androgen receptor axis-targeted therapy agents (ARATAs) become insignificant when discussed separately in terms of subgroups of flare.

That PSA increases after a specific stage of therapy is widely accepted as a factor for mCRPC deterioration [26, 37]. However, we acknowledge that flare might interfere with this outcome. Although PSA increased at 3 months after therapy, the OS was significantly poor only in the patients without flare but not in flare group.

The same is true of ARATAs. Abiraterone acetate and enzalutamide are classified as ARATAs although their pathways differ, and they have been confirmed as contributors to treatment for mCRPC [38, 39]. The 2-year OS rates did not distinctly differ among patients with flare, regardless of ARATAs. When the survival rate is significantly low among patients without BSI flare, ARATAs can elevate the OS to a level similar to that of patients with BSI flare. However, some studies using PET/CT imaging have not identified bone flare in patients with mCRPC treated with enzalutamide [15, 40], whereas a large-scale study detected them in 18.1–27.5% patients [36]. The present study found no difference in the incidence of flare between abiraterone acetate and enzalutamide therapy. We speculate that sample size, different imaging methods, and pretreatments could be the reasons for the diverse conclusions.

The Cox proportional hazards analysis also supported this finding. Our univariable analysis indicated that elevated baseline serum values for the biomarkers ALP, BAP, PSA, and the BSI were prognostic for the 2-year OS of patients without flare. Higher BSI and PSA were identified as independent risk factors and generally agreed with those previously published [6, 15, 30, 33, 35, 40, 43]. However, none of these variables were associated with the 2-year OS in patients with flare. Although liver or lung metastases are apparently significant, the low proportion of patients (4 [15%] of 27) rendered this notion questionable. We suppose that flare results in a more stable prognosis that is less susceptible to outside influences for patients with mCRPC.

### Limitation

This study was limited by the relatively small patient cohort derived from a subset of the multicenter PROSTAT-BSI study. Not all patients had access to bone imaging every 3 months, and some had no subsequent tracking data. Further validation of more patients is needed, and blood should be analyzed within 3 months to further understand the relationship between bone flare and serum biomarkers. Furthermore, all our results were derived from patients treated with docetaxel. For a more comprehensive understanding, other treatments that induce flare, such as ^233^Ra and ARATAs, should be analyzed to enhance the credibility of our results.

## Conclusion

We showed here that the bone flare phenomenon indicated a favorable prognosis for patients in mCRPC treated with docetaxel. The predictive effects of other variables on OS, such as abiraterone or/and enzalutamide therapy, increased PSA, and the BSI that was prevalent in patients without flare were diminished in the presence of flare. Follow-up bone scintigraphy after 3 months of drug administration should be recommended to assess the prognosis of patients and provide guidance for further medical regimens.

## Data Availability

The use of datasets generated and analyzed during the current study is approved for paticipated institutions by Ethics Committee. However, it could be available from the corresponding author on reasonable request.

## Abbreviations

^99m^Tc-MDP: ^99M^Tc-methylene diphosphonate
ACD: All-caused death
BSI: Bone scan index
CI: Confidence interval
HR: Hazard ratio
mCRPC: Metastatic castration-resistant prostate cancer
OS: Overall survival
PCaD: Prostate cancer-caused death
PFS: Progression-free survival
PROSTAT‐BSI: Prostatic Cancer Registry of standard hormonal and chemotherapy using Bone Scan Index
